# Supplementary material for: End Sequence Analysis Toolkit (ESAT) expands the extractable information from single-cell RNA-seq data
Source: Genome Res. 2016 Oct;26(10):1397–410. doi: 10.1101/gr.207902.116 (PMC5052061; doi:10.1101/gr.207902.116)
Supplement: Supplemental Material [file supp_gr.207902.116_Supp_Materials.tar.gz › ESAT-esat_v0.1/doc/html/classumms_1_1core_1_1annotation_1_1_single_interval.html]

ESAT: umms.core.annotation.SingleInterval Class Reference


ESAT


- Main Page
- Related Pages
- Packages
- Classes
- Files

- Class List
- Class Index
- Class Hierarchy
- Class Members

All Classes Namespaces Files Functions Variables Enumerator Pages

- umms
- core
- annotation
- SingleInterval

Public Member Functions |
List of all members

umms.core.annotation.SingleInterval Class Reference

Inheritance diagram for umms.core.annotation.SingleInterval:

[legend]

Collaboration diagram for umms.core.annotation.SingleInterval:

[legend]

|  |  |
| --- | --- |
| Public Member Functions | |
|  | SingleInterval () |
|  | |
|  | SingleInterval (int start, int end) |
|  | |
|  | SingleInterval (Interval other) |
|  | |
| boolean | overlaps (SingleInterval other) |
|  | |
| boolean | contains (SingleInterval other) |
|  | |
| int | getDistanceTo (SingleInterval other) |
|  | |
| SingleInterval | intersect (SingleInterval other) |
|  | |
| SingleInterval | union (SingleInterval other) |
|  | |
| int | compareTo (SingleInterval other) |
|  | |
| boolean | equals (SingleInterval other) |
|  | |
| String | toString () |
|  | |

## Detailed Description

Author
:   engreitz This class represents a single interval on a integer coordinate space.

## Constructor & Destructor Documentation

|  |  |  |  |  |
| --- | --- | --- | --- | --- |
| umms.core.annotation.SingleInterval.SingleInterval | ( |  | ) |  |

Here is the caller graph for this function:

|  |  |  |  |
| --- | --- | --- | --- |
| umms.core.annotation.SingleInterval.SingleInterval | ( | int | *start*, |
|  |  | int | *end* |
|  | ) |  |  |

|  |  |  |  |  |  |
| --- | --- | --- | --- | --- | --- |
| umms.core.annotation.SingleInterval.SingleInterval | ( | Interval | *other* | ) |  |

Here is the call graph for this function:

## Member Function Documentation

|  |  |  |  |  |  |
| --- | --- | --- | --- | --- | --- |
| int umms.core.annotation.SingleInterval.compareTo | ( | SingleInterval | *other* | ) |  |

Here is the call graph for this function:

Here is the caller graph for this function:

|  |  |  |  |  |  |
| --- | --- | --- | --- | --- | --- |
| boolean umms.core.annotation.SingleInterval.contains | ( | SingleInterval | *other* | ) |  |

Here is the call graph for this function:

|  |  |  |  |  |  |
| --- | --- | --- | --- | --- | --- |
| boolean umms.core.annotation.SingleInterval.equals | ( | SingleInterval | *other* | ) |  |

Here is the call graph for this function:

|  |  |  |  |  |  |
| --- | --- | --- | --- | --- | --- |
| int umms.core.annotation.SingleInterval.getDistanceTo | ( | SingleInterval | *other* | ) |  |

Parameters
:   |  |  |
    | --- | --- |
    | other |  |

Returns
:   0 if overlapping, otherwise the minimum absolute distance from one to the other.

Here is the call graph for this function:

|  |  |  |  |  |  |
| --- | --- | --- | --- | --- | --- |
| SingleInterval umms.core.annotation.SingleInterval.intersect | ( | SingleInterval | *other* | ) |  |

Parameters
:   |  |  |
    | --- | --- |
    | other |  |

Returns
:   New interval of intersection; if they don't intersect, then returns null

Here is the call graph for this function:

|  |  |  |  |  |  |
| --- | --- | --- | --- | --- | --- |
| boolean umms.core.annotation.SingleInterval.overlaps | ( | SingleInterval | *other* | ) |  |

Here is the caller graph for this function:

|  |  |  |  |  |
| --- | --- | --- | --- | --- |
| String umms.core.annotation.SingleInterval.toString | ( |  | ) |  |

Here is the call graph for this function:

|  |  |  |  |  |  |
| --- | --- | --- | --- | --- | --- |
| SingleInterval umms.core.annotation.SingleInterval.union | ( | SingleInterval | *other* | ) |  |

Parameters
:   |  |  |
    | --- | --- |
    | other |  |

Returns
:   New interval spanning both intervals, including the space between them if they don't overlap

Here is the call graph for this function:

---

The documentation for this class was generated from the following file:

- java/umms/core/annotation/SingleInterval.java


---

Generated on Fri Aug 15 2014 15:11:10 for ESAT by  

 1.8.7
